# Spontaneous Splenic Rupture in a Chronic Hemodialysis Patient: A Case Report

**DOI:** 10.7759/cureus.97450

**Published:** 2025-11-21

**Authors:** Adib Remmal, Taha Iken, Asmae Oulad Amar, Bouchra Dahmani, Siham Alaoui Rachidi

**Affiliations:** 1 Radiology, Mohammed VI University Hospital Center, Tangier, MAR; 2 Radiology, Faculty of Medicine and Pharmacy, Abdelmalek Essaadi University, Tangier, MAR

**Keywords:** chronic hemodialysis patients, contrast extravasation, hemodialysis, intradialytic hypotension, lupus nephropathy, splenectomy, spontaneous splenic rupture, systemic lupus erythematosus

## Abstract

Intradialytic hypotension (IDH) is a common and potentially severe complication of hemodialysis, usually caused by poor ultrafiltration prescriptions or inappropriate dry weight determination. Nevertheless, its relation with dialysis-independent causes cannot be ruled out. This case report presents a 35-year-old woman undergoing chronic hemodialysis for lupus nephropathy, who experienced IDH secondary to an unusual manifestation of spontaneous splenic rupture (SSR). We report the clinical presentation, workup, and management of this infrequent but potentially life-threatening syndrome.

## Introduction

One of the most frequent and dangerous side effects of hemodialysis is intradialytic hypotension (IDH). It affects approximately 20-30% of outpatient dialysis sessions and is associated with increased cardiovascular morbidity and mortality, as well as decreased quality of life [1,2]. IDH is typically caused by a combination of factors such as excessive ultrafiltration, inadequate vascular refilling, autonomic dysfunction, and failure to accurately estimate the patient's dry weight [3]. Dialysis-related causes, including overly aggressive fluid removal, incorrect dialysate composition, and reduced cardiac reserve, are often implicated in the development of this syndrome.

However, while dialysis-related factors are usually the primary suspects, clinicians must remain vigilant for non-dialysis-related causes of acute hypotension. Rarely, the clinical presentation of IDH may be mimicked or exacerbated by spontaneous visceral organ rupture, especially of the spleen. The rare but potentially fatal condition known as spontaneous splenic rupture (SSR) can manifest as hypotension, abdominal pain, and symptoms of hypovolemic shock. It has been linked to systemic inflammatory diseases such as systemic lupus erythematosus (SLE), infections, and cancers [4-6].

Here, we report a case of a 35-year-old woman with lupus nephropathy undergoing maintenance hemodialysis who developed IDH, which was subsequently determined to be caused by SSR. This case emphasizes the importance of considering uncommon, non-dialysis-related causes when diagnosing IDH, especially in patients with underlying systemic diseases.

## Case presentation

A 35-year-old woman with end-stage kidney disease on maintenance hemodialysis presented to the emergency department following an intradialytic episode of hypotension, chills, and left lumbar pain. Her dialysis session had been interrupted due to hemodynamic instability and discomfort.

On physical examination, left-sided lumbar pain and diffuse abdominal tenderness were noted. Her laboratory workup yielded the hemoglobin of 12.5 g/dL, leukocytosis (13,220/mm³) with neutrophil predominance (94,000/mm³), and C-reactive protein (CRP) of 83 mg/L. See Table 1 for laboratory workup.

**Table 1:** Laboratory workup

Test	Result	Unit of Measurement	Normal Reference Range	Comments
Hemoglobin (Hb)	12.5	g/dL	12–16 g/dL (women)	Within normal limits
Leukocytes (WBC count)	13,220	/mm³	4,000–10,000 /mm³	Elevated → Leukocytosis
Neutrophils (PNN)	Predominance	—	40–70%	Suggests neutrophilic leukocytosis
Platelets (PLT)	94,000	/mm³	150,000–450,000 /mm³	Thrombocytopenia
C-reactive protein (CRP)	83	mg/L	<5 mg/L	Elevated → Significant inflammation

**Figure 1:**
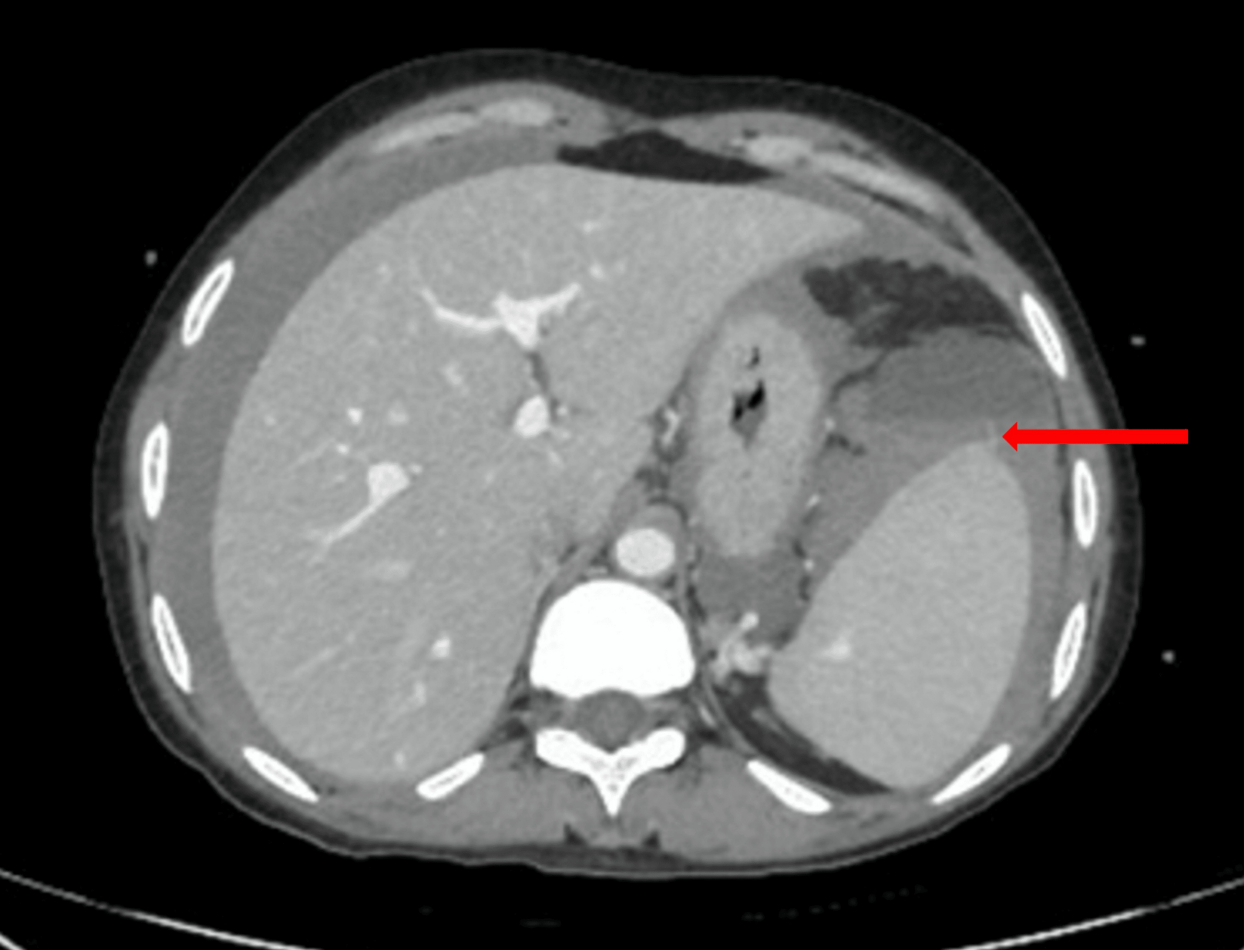
Extravasation seen on CT (red arrow) with hemoperitoneum Axial image of an abdominal CT scan showing active leakage (extravasation) from the spleen, with hemoperitoneum.

The patient was treated empirically with antibiotics since the clinical presentation suggested a likely infection. She was also vasopressor-supported and fluid resuscitated. Figure 1 denotes vascular injury with differential densities in ascitic fluid, indicating active hemoperitoneum.

**Figure 2:**
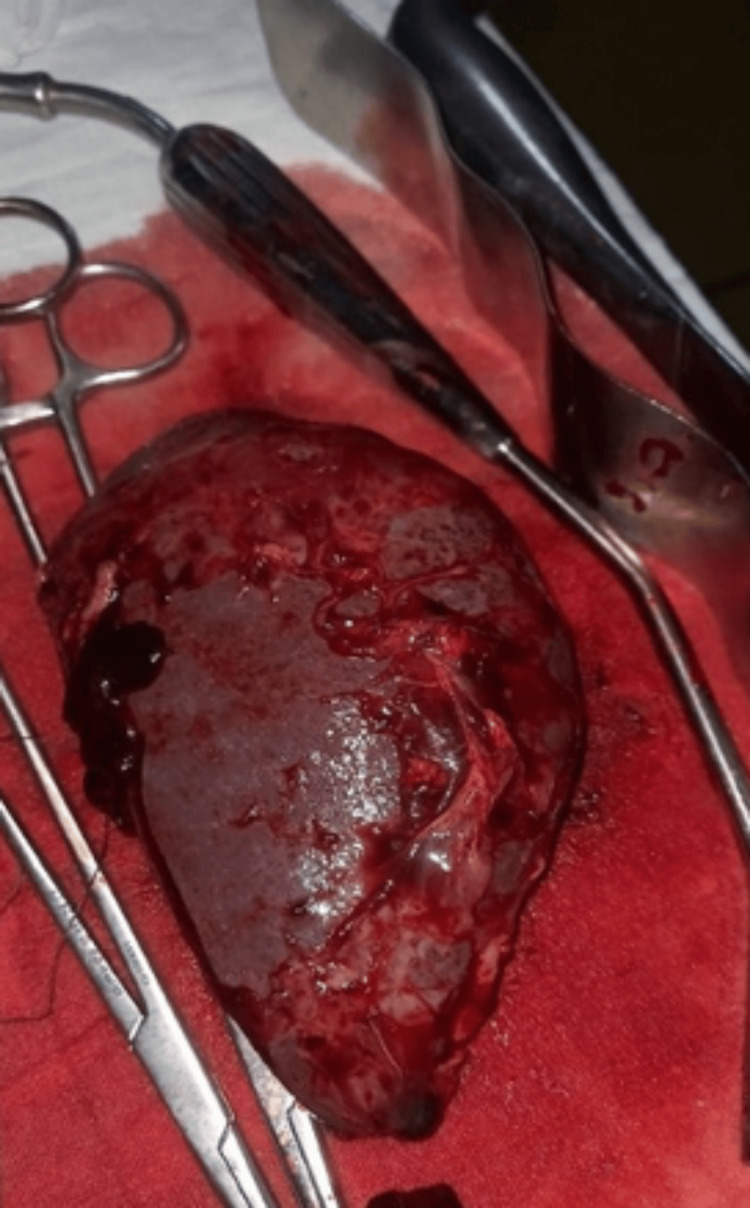
Postoperative spleen Surgical exploration of the spleen demonstrating a superficial rupture corresponding with the extravasation seen on CT.

The patient was transferred to the operating room promptly, where splenectomy was performed to control the hemorrhage. Histopathological examination of the spleen was discovered to be a fibro-congestive and hyperplastic spleen without malignancy (Figure 2).

Postoperatively, the patient recovered gradually, with normalization of her hemodynamics and resolution of symptoms. Exclusion studies for other causes of the splenic rupture were done during follow-up, and, as anticipated, lupus nephropathy was confirmed to be the underlying causative etiology.

## Discussion

SSR is a rare but potentially fatal complication in patients with SLE. Splenomegaly due to the chronic inflammatory process of the disease is a common cause of splenic rupture. Fibrosis, hyperplasia, and congestion of the spleen, which are frequently encountered in SLE patients, enhance its vulnerability to rupture, particularly when anticoagulation therapy is administered - a condition often encountered in hemodialysis patients [5,7].

SSR can present as acute, severe abdominal pain with a strong association with shock and hemodynamic instability. The clinical presentation of SSR can mimic other common complications in dialysis patients, including sepsis, hemolysis, or vascular complications; hence, early diagnosis can be challenging. Imaging modalities such as abdominal ultrasound and angiography are crucial for diagnosis [8]. In this case, the patient’s clinical deterioration during dialysis and imaging findings led to the diagnosis of rupture.

Although the exact mechanism by which SSR occurs in SLE patients is not fully understood, it is believed that the synergy between splenomegaly and fragility of the splenic vasculature increases the risk of rupture, especially in the context of anticoagulation therapy used during hemodialysis [4,8]. This case highlights the importance of considering non-dialysis-related etiologies when evaluating patients with IDH, particularly those with systemic inflammatory diseases such as lupus.

Management of SSR typically involves surgical intervention to control hemorrhage and stabilize the patient. Splenectomy remains the treatment of choice in hemodynamically unstable patients. Early diagnosis and prompt surgical intervention are crucial to improving outcomes. In stable patients, conservative care with close monitoring can be attempted, although splenectomy is generally recommended to prevent recurrent rupture and control ongoing hemorrhage [9].

In our patient, splenectomy successfully controlled the bleeding, and postoperative recovery was smooth. Given the rarity of SSR in SLE, this case underscores the need for physicians to maintain a high degree of suspicion for less common causes of IDH, especially in patients with known risk factors such as lupus nephropathy.

## Conclusions

SSR is a rare but life-threatening condition in SLE, especially when such patients are undergoing hemodialysis. The presentation of SSR can be unusual, for instance, as intradialytic hypotension, and is obscured by other complications. In this case, IDH initiated the diagnostic process that revealed SSR, complicated further by anticoagulation therapy during dialysis. Early recognition by appropriate imaging and early surgical treatment are important to improve patient outcomes. Clinicians need to keep in mind the potential for non-dialysis-related complications of hypotension and other conditions in chronic dialysis patients, particularly with chronic underlying systemic disease such as lupus.
